# Blinding and sham control methods in trials of physical, psychological, and self-management interventions for pain (article I): a systematic review and description of methods

**DOI:** 10.1097/j.pain.0000000000002723

**Published:** 2022-07-11

**Authors:** David Hohenschurz-Schmidt, Jerry Draper-Rodi, Lene Vase, Whitney Scott, Alison McGregor, Nadia Soliman, Andrew MacMillan, Axel Olivier, Cybill Ann Cherian, Daniel Corcoran, Hilary Abbey, Sascha Freigang, Jessica Chan, Jules Phalip, Lea Nørgaard Sørensen, Maite Delafin, Margarida Baptista, Naomi R. Medforth, Nuria Ruffini, Stephanie Skøtt Andresen, Sylvain Ytier, Dorota Ali, Harriet Hobday, Anak Agung Ngurah Agung Adhiyoga Santosa, Jan Vollert, Andrew S.C. Rice

**Affiliations:** a Pain Research, Department of Surgery and Cancer, Faculty of Medicine, Imperial College, Chelsea, London, United Kingdom; b Research Centre, University College of Osteopathy, London, United Kingdom; c Section for Psychology and Neuroscience, Department of Psychology and Behavioural Sciences, Aarhus University, Aarhus C, Denmark; d Health Psychology Section, Department of Psychology, Institute of Psychiatry, Psychology and Neuroscience, King's College London, London, United Kingdom; e INPUT Pain Management Unit, Guy's and St Thomas' NHS Foundation Trust, London, United Kingdom; f Human Performance Group, Department of Surgery and Cancer, Faculty of Medicine, Imperial College, London, United Kingdom; g Faculty of Medicine, Imperial College London, London, United Kingdom; h Chemical Engineering Department, Khalifa University, Abu Dhabi, United Arab Emirates; i West Ballina, Australia; j Department of Neurosurgery, Medical University Graz, Graz, Austria; k Résidence les Estrangers, La Bourboule, France; l Department of Occupational Medicine, Danish Ramazzini Centre, Aarhus University Hospital, Aarhus, Denmark; m The Penn Clinic, Hertfordshire, Hatfield, United Kingdom; n Department of Psychology, Wolfson Centre for Age Related Diseases, Institute of Psychiatry, Psychology and Neuroscience, King's College London, London, United Kingdom; o London, United Kingdom; p National Centre Germany, Foundation C.O.M.E. Collaboration, Berlin, Germany; q Department of Psychology and Behavioural Sciences, Aarhus University, Aarhus C, Denmark; r Vevey, Switzerland; s Genetic and Developmental Psychiatry Centre, Institute of Psychiatry, Psychology and Neuroscience, King's College London, London, United Kingdom; t Institute of Psychiatry, Psychology and Neuroscience, King's College London, London, United Kingdom; u Denpasar, Indonesia; v Division of Neurological Pain Research and Therapy, Department of Neurology, University Hospital of Schleswig-Holstein, Kiel, Germany; w Neurophysiology, Mannheim Center of Translational Neuroscience (MCTN), Medical Faculty Mannheim, Heidelberg University, Heidelberg, Germany; x Department of Anaesthesiology, Intensive Care and Pain Medicine, University Hospital Muenster, Muenster, Germany

**Keywords:** Randomised controlled trials, Placebos, Placebo effect, Control groups, Systematic review, Physical therapy modalities, Rehabilitation, Psychotherapy

## Abstract

Blinding is challenging in randomised controlled trials of physical, psychological, and self-management therapies for pain, mainly because of their complex and participatory nature. To develop standards for the design, implementation, and reporting of control interventions in efficacy and mechanistic trials, a systematic overview of currently used sham interventions and other blinding methods was required. Twelve databases were searched for placebo or sham-controlled randomised clinical trials of physical, psychological, and self-management treatments in a clinical pain population. Screening and data extraction were performed in duplicate, and trial features, description of control methods, and their similarity to the active intervention under investigation were extracted (protocol registration ID: CRD42020206590). The review included 198 unique control interventions, published between 2008 and December 2021. Most trials studied people with chronic pain, and more than half were manual therapy trials. The described control interventions ranged from clearly modelled based on the active treatment to largely dissimilar control interventions. Similarity between control and active interventions was more frequent for certain aspects (eg, duration and frequency of treatments) than others (eg, physical treatment procedures and patient sensory experiences). We also provide an overview of additional, potentially useful methods to enhance blinding, as well as the reporting of processes involved in developing control interventions. A comprehensive picture of prevalent blinding methods is provided, including a detailed assessment of the resemblance between active and control interventions. These findings can inform future developments of control interventions in efficacy and mechanistic trials and best-practice recommendations.

## Abstract 2

Supplemental Digital Content is Available in the Text.

## 1. Introduction

The opioid crisis and the insufficiency of many widely used pain treatments highlight the need for nonpharmacological and nonsurgical pain therapies.^1,95,98,119^ Such therapies include cognitive-behavioural approaches, exercise and rehabilitation, manual therapies, acupuncture, mind–body techniques such as yoga, devices such as ultrasound and light therapy, electrical therapies, and education; referred to as physical, psychological, and self-management therapies (PPS) from here on. Current guidelines recommend various nondrug therapies as a first-line treatment for low back and chronic musculoskeletal pain.^24,98,126^ However, most recommendations are based on low-quality or moderate-quality evidence,^139^ a widespread concern in PPS interventions.^7,46,49,64,67,101,137^ A lack of high-quality research means that the role of many of these therapies in the prevention, treatment, and management of pain is unclear. This lack of high-quality data is partly because of methodological difficulties specific to efficacy and mechanistic trials of PPS for pain, mainly centred around issues of placebo control and blinding.^32,33,103^

Placebo interventions in clinical trials are conceptualised as “*a control intervention with similar appearance as the experimental treatment, but void of the components in the experimental intervention whose effects the trial is designed to evaluate.*”^88^ Recognising that, in nonpharmacological trials, such control interventions are not usually “inert,” the term “sham intervention” is used in this context.^85,113^ Sham-controlling a trial is desirable when specific and context-related treatment effects are to be distinguished (efficacy trials), to test the effects of particular treatment components (mechanistic trials) and to reduce bias by allowing for blinding of participants and ideally researchers and clinical personnel.^78,133^ Blinding or masking refers to the attempt to conceal group allocation or study hypotheses from study participants, therapists, or researchers^58^ so that expectation effects and manipulation of trial procedures do not undermine internal validity.^159^ Notably, the prominent role of blinding in clinical trials is debated.^9,59,117,162^ Irrespectively, there are many scenarios in which controlling for placebo effects is considered important, including pain research because of the arguable susceptibility of subjective symptoms to placebo^154,161,168^ and to address the question of whether treatments are efficacious beyond context-dependent effects.^9,60,93,131^

In nonpharmacological randomised controlled trials (RCTs), sham-controlling is more challenging than in drug studies and blinding is more difficult^7,33^ because care providers are often an integral part of the treatment and cannot be blinded. The complex participatory nature of these interventions often precludes the design of control conditions that feel authentic to patients. Notable exceptions are device-delivered therapies, where the sham simply involves detuned devices^31^; surgery where much work on sham controls is conducted and which benefits from general anaesthesia for blinding^22,53,71,160^; and acupuncture, using needling in nonacupuncture points or non- or low-level penetrating sham needles, resulting in reasonable opportunity for participant blinding.^34,36,149^ These therapies are therefore not discussed here.

In all other areas of PPS interventions, however, unifying criteria for the development, implementation, and reporting of dedicated control interventions for efficacy and mechanistic trials are lacking. Instead, trials of cognitive-behavioural interventions, rehabilitation, exercise, mind–body therapies, and physical and manual therapies often resort to waitlist controls as comparators or different therapeutic modalities, arguing that “blinding is not possible.”^33^ However, comparisons with no-treatment arms lead to exaggerated effect sizes,^61,115^ and comparative effectiveness designs commonly address different research questions than efficacy and mechanistic trials.^56,65,171^ In 2007, it was found that sham interventions in nonpharmacological RCTs did not frequently resemble the experimental treatment,^31^ arguably increasing unblinding risk. In particular, nonmatching controls do not reliably distinguish specific treatment effects from context-dependent effects.^31,125,149^ The concept of “structural equivalence” was proposed to enhance matching between control and experimental treatments.^21^ Furthermore, a range of features for which conditions should be similar or even “indistinguishable” was introduced, from the number of treatments, to procedural steps in the application of interventions, to the personal interactions with therapists and staff.^16,35,36,62,77,125^ Recently, reporting guidelines for sham interventions were published, encompassing many of these features.^86^ There is, however, no evidence-based and unifying framework that specifies which theoretical, practical, and ethical considerations should guide researchers in the development, implementation, and evaluation of control interventions in efficacy and mechanistic trials.

To inform such guidance applicable across PPS interventions, a comprehensive overview of currently used sham interventions and other methods to enhance blinding is needed. This systematic review of methods aimed to identify common and less common control intervention designs in RCTs of PPS for a clinical population of patients with pain. Furthermore, we provide a detailed similarity assessment across 25 features for which matching between control and experimental treatments has been said to be important, allowing for comparisons between therapy types. In addition, we identify studies that report on blinding effectiveness and control intervention validation studies. In a parallel publication,^81^ the potential impact of these control methods on trial results are formally examined.

## 2. Methods

A systematic review of methods was conducted and is reported according to the PRISMA 2020 statement.^121^

### 2.1. Protocol and registration

The protocol was registered with the International Prospective Register of Systematic Reviews (PROSPERO, registration ID: CRD42020206590). The material here presented is the first part of this protocol (including the results of the following analyses: descriptives and subgroups, trial reporting, degree of similarity between control intervention and treatment, blinding indices); a second article includes the meta-analysis.^81^

### 2.2. Eligibility criteria

This review included RCTs of PPS interventions for adults living with pain, irrespective of sex, underlying pathology or pain severity and duration. At least 1 pain-related primary outcome measure had to be reported. Physical, psychological, and self-management included all forms of manual and physical therapy; exercise and rehabilitation therapy; conversation-based and psychological therapies; body–mind, spiritual, religious, and other nonmaterial healing practices; web-based therapies; relaxation; and educational interventions (the latter 2 were classified as “self-management” here). To be eligible, trials had to use a sham control intervention (or “attention” or “placebo control” Table 1).

**Table 1:** Eligibility criteria for inclusion into the systematic review.

Population	Interventions	Comparator	Outcomes	Design	Timeframe
Included:					
Any pain Adults	Physical therapies Psychological interventions Self-management	Placebo Sham Attention controls	Pain-related primary	RCTs	January 2008-24 November 2021
Excluded:					
Experimental pain	Surgery Devices Acupuncture Meridian therapy	Other designated intervention groups (comparative effectiveness) Waitlist or no treatment Treatment as usual		Pilot and feasibility RCTs (as defined by the primary study authors)	

RCT, randomised controlled trial.

Excluded were studies where pharmacological or drug interventions formed the mainstay of treatment and studies of surgical or otherwise invasive interventions. Furthermore, all therapies relying on the permanent introduction of some form of matter into the body were excluded. Owing to specific considerations and solutions to the sham-control problem in device-based and needle-based therapies,^31,34,36,149^ studies from these categories were also not eligible. Implanted and externally applied devices, all acupuncture modalities, and therapies based on assumed reflex points or energy meridians were excluded.

We excluded nonrandomised studies, observational studies, cross-sectional studies, case-control, case-series, and case-report studies. Pilot or feasibility RCTs were excluded, except for validation studies assessing the sham interventions in an adult population of patients with pain, irrespective of using pain-related outcomes.

For included studies, trial protocols were consulted where available and required for additional method information. The first reporting guideline for nonpharmacological therapy trials was published in February 2008.^32^ Therefore, this review systematically assessed studies published from 2008 onwards.

### 2.3. Data sources

The following databases were searched from 2008 to November 2021 (initial search conducted on June 23, 2020, then updated; latest search: November 24, 2021): MEDLINE, Embase, PsychInfo, the Cochrane Database of Systematic Reviews, the Cochrane Central Register of Controlled Trials (CENTRAL), NIH ClinicalTrials.gov, AMED (Allied and Complementary Medicine), CINAHL (nursing and allied health), the Physiotherapy Evidence Database (pedro.org.au), ostmed.dr (ostmed-dr.oclc.org), Osteopathic Research Web (osteopathic-research.com), and the Index to Chiropractic Literature (chiroindex.org).

### 2.4. Search strategy

The search strategy was built around the following keywords, developed based on existing literature and with database experts, and is provided in full for each database in the digital supplemental materials (supplemental digital content 1, spreadsheet including search results, available at http://links.lww.com/PAIN/B671).

Pain OR painful conditions AND Physical, Psychological, Self-management therapies (specific therapy and technique names) AND placebo control OR sham control OR attention control AND controlled clinical trials. Limit: 2008 to present.

### 2.5. Study selection

Eligibility screening was performed in duplicate by 2 independent reviewers drawn from a pool of specifically trained research contributors. Disagreements were resolved by a third reviewer. The screening was first performed based on study title and abstract. Full-text eligibility was assessed in a second step.

### 2.6. Data extraction

The data extraction process also required a minimum of 2 independent reviewers. Discrepancies were resolved through discussion or by a third independent reviewer.

Publications reporting multiple sham controls were extracted independently for each pair of intervention and sham, with data from an active intervention arm used twice for comparisons with control interventions if required. Where a single placebo control group acted as a comparator for multiple active interventions, data were extracted from the active intervention that most resembled the control intervention.

Data extraction was trialled using a sample of potentially eligible studies. Data extraction was performed by volunteer reviewers with at least a Masters-level qualification in a biomedical subject and a minimum time commitment of 3 hours per week on the project. Training in systematic review methods, trial design, and the use of online platforms was provided by the lead investigator (D.H.-S.) before starting data extraction. The results of the pilot testing informed the final approach to data extraction, with detailed annotations for extraction items available to reviewers and reliability monitored throughout.^79^

Data extraction domains were bibliographic data, general study design, trial reporting, sham control and blinding methods, trial results, and risk of bias (the latter 2 are reported in a separate publication^81^).

### 2.7. Data analysis

#### 2.7.1. Descriptive analysis and subgroups

This publication reports the qualitative part of the data synthesis, providing an overview of blinding methods used in the field of PPS therapies for pain, including basic description of sham interventions, their development and reported rationale, the similarity between control and active interventions, compliance with relevant reporting guidelines (notably the intervention description and blinding items of the Consolidated Standards of Reporting Trials (CONSORT), extension for nonpharmacological trials,^30^ and reports of blinding effectiveness. Apart from providing these data for the entire sample, data were subgrouped by therapy type where appropriate. Given the size and complexity of this review, the results of a formal risk of bias assessment^138^ and analyses including pain-related and other outcome data are reported in a parallel article^81^ to ensure sufficient interpretation.

### 2.8. Meta-analysis: similarity index and ratings

**Figure 1.**
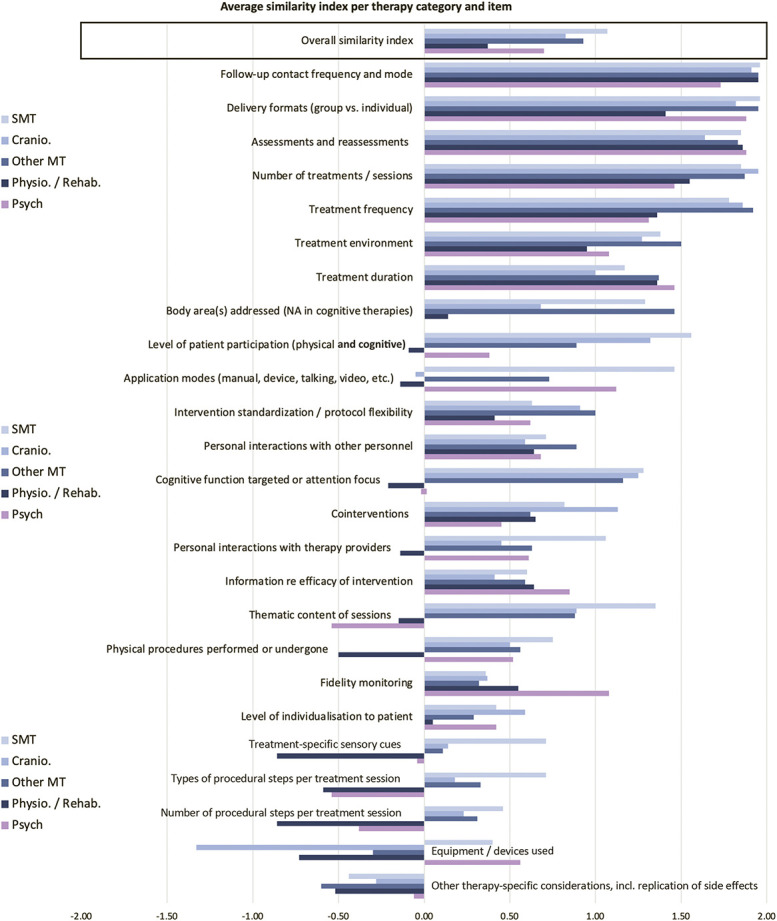
PRISMA flow diagram of the systematic search and selection process. The total number of included studies differs from the number of test treatment or control comparisons because of trials with multiple sham controls or single sham controls used as comparators for multiple active arms. In total, 198 unique sham interventions were included, one of which used twice for a comparison with an active arm and reported in 194 publications. A complete search strings per database and a list of all studies excluded at the full-text screening stage are provided in the supplementary digital content 1, available at http://links.lww.com/PAIN/B671. RCT, randomised controlled trial.

**Figure 2.**
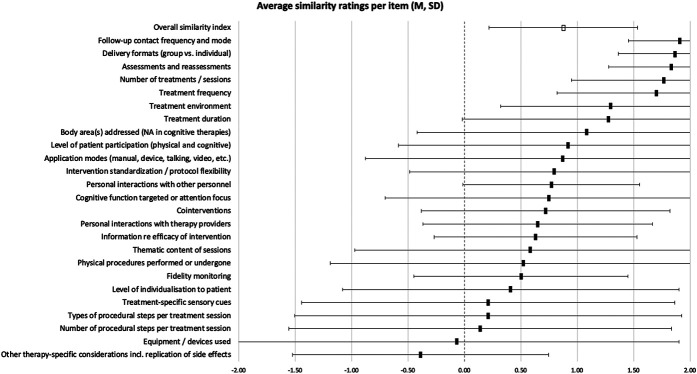
Similarity between tested intervention and placebo control intervention for all included studies. Items were rated if applicable to individual studies (N provided in supplemental digital content 3, available at http://links.lww.com/PAIN/B673), with the following possible ratings and corresponding numerical values: yes = 2, probably yes = 1, not reported = 0, probably no = −1, no = −2. Full squares indicate mean and SDs are provided as error bars. The empty square represents the overall mean across all items and studies. N = 198.

A high degree of similarity between control and test intervention is commonly assumed to be a desirable feature of controlled efficacy and mechanistic trial designs.^16,21,36,62,77,108,125,146^ While some authors have used concepts of “indistinguishability” and “structural equivalence” to denote different levels of similarity,^21,108,125^ we drew on such work to define 25 features across which control and treatment interventions may be compared. Assessed features are listed in Figures 1 and 2 and were based on a review of the following pertinent literature:^16,21,30,35,36,41,48,62,77,87,125,128,143,146^

Similarity ratings were based on the reviewers' evaluation of how similar individual items were between active and sham interventions. Specifically, “yes” (similar) and “no” (dissimilar) evaluations were rated as 2 and −2, respectively. “Probably yes” and “probably no” were awarded 1 and −1 points, and 0 points were given for each item that could not be rated because of insufficient information. Nonapplicable items were not rated. In addition, each trial's total ratings were divided by the number of rated items to produce a single value, encompassing similarity across all applicable items. This is for illustrative purposes only because it is unclear whether all items can be weighted equally. Values of the item-specific group averages and the overall similarity average range from −2 (dissimilar across all studies or rated items) to 2 (similar). Data for individual items and the overall index were synthesised as means and standard deviations for each therapy group.

### 2.9. Meta-analysis: reports of blinding success and blinding indices

During data extraction, we identified studies indicating the effectiveness of the used blinding methods, for example, by having patients guess their group allocation or rate the treatment credibility. Methodological detail and self-reported blinding effectiveness of these studies are reported descriptively. Where group guesses were reported in a manner that allowed for the calculation of Bang's blinding index, the index was calculated for active and control groups individually.^20^ Specifically, absolute numbers or the percentages of participants per group guessing their allocation correctly, incorrectly, or being unsure were extracted. A ratio of Bang's blinding index was calculated as Hedges g for each comparison between test and control group.^50^

## 3. Results

### 3.1. Sample description

The flowchart in Figure 1 provides an overview of the study selection process and Table 2 of the reviewed trials' characteristics. Data were extracted from 194 publications (plus protocols where available), reporting 198 sham control interventions. Manual therapy trials dominated, followed by psychological and rehabilitation trials. Most commonly, patients with musculoskeletal pain were treated.

**Table 2:** Overview of included studies.

	n of studies	%
Therapy types		
Manual therapy with spinal manipulation	48	24.2
Craniosacral therapy and gentle myofascial release	22	11.1
Other manual therapy	64	32.3
Rehabilitation or physiotherapy	22	11.1
Self-management	5	2.5
Cognitive-behavioural and other psychotherapy	27	13.6
Spiritual or energetic or esoteric healing	8	4.0
Other	2	1.0
Intervention complexity†		
Simple	112	56.6
Complex	86	43.4
Pain descriptor		
Musculoskeletal pain	121	61.1
Diffuse chronic pain	18	9.1
Cancer-related pain	6	3.0
Visceral pain	5	2.5
Neuropathic pain	5	2.5
Pregnancy-related pain	1	0.5
Not specified	1	0.5

	Median	Q1/Q3
Sample size at randomization		
Overall sample size (all trial arms combined)	64	40/101
Sample size per trial arm (only groups included in review)	27	19.5/46

	n	%
Registered trial protocol available		
Registered	114	57.9
Group design		
Parallel group	163	92.9
Cross-over	14	7.1
No. of study conditions per trial*		
2	139	71.6
3	46	23.7
4	8	4.1
5	1	0.5
6	1	0.5
Additional nonsham comparators included		
Active comparator (comparative effectiveness) ^±^	29	14.9
No treatment or waitlist	20	10.3
Usual care/treatment as usual	13	6.7

The types of therapies, intervention complexity, and pain population are provided for the entire sample. Special cases: In 1 trial, data from the active intervention group were used twice to compare it with 2 different sham controls: Bialosky et al. (2014) used a “standard” and an “enhanced” sham control. Three publications reported more than 1 trial: D'Souza et al. (2008) studied 2 groups with different types of headaches, and the publication of Assefi et al. (2008) included 2 active interventions and a matching sham control each. Finally, Sharpe et al. (2012) reported 2 trials in a single publication, which were treated entirely independently here. In general, only patients who informed the present analyses are counted in this table; patients were not counted twice, and analyses of reporting refer to individual trials.

* Each intervention or sham intervention was counted, irrespective of whether the trial was a single-arm cross-over trial. ^±^ So-called attention controls were not counted as active comparator, only experimental conditions that were clearly assessed because they were deemed potentially effective alternatives (comparative effectiveness intention).

† Intervention complexity: Single-step or single-technique interventions were judged as “simple,” irrespective of how often these were applied, and others as complex. N = 194 publications with 198 comparisons between treatment and a sham control.

### 3.2. Validation studies

We included 8 validation studies of sham control interventions tested in patients with pain.^37,48,55,73,76,94,112,150^

### 3.3. Placebo and sham control intervention designs

The CONSORT statement asks researchers to describe “[t]he interventions for each group with sufficient details to allow replication, including how and when they were actually administered.”^30,133^ In our sample of 198 sham control interventions, 67% complied with this reporting item and provided a description of *the control intervention* while 77% did for the experimental treatment.

Table 3 provides an overview of the main features of all reviewed sham interventions, categorised by therapy type (see supplemental digital content 2 for table providing classification at study level, available at http://links.lww.com/PAIN/B672).

**Table 3:** Overview of used placebo control interventions per therapy type.

Therapy type	Total N	Placebo control or sham interventions	N	%
Manual therapy with spinal manipulation	49	Manual, simulated manoeuvre	30	61.2
		Manual, soft touch	9	18.4
		Manual, technique to different area	1	2.0
		Disabled device	6	12.2
		Rest time-control	1	2.0
		Other (therapist attention, general anaesthesia)	2	4.1
Craniosacral therapy and gentle myofascial release	22	Manual, simulated manoeuvre	2	9.1
		Manual, soft touch	8	36.4
		Disabled device	10	45.5
		Other (low-strength static magnets, active joint movement)	2	9.1
Other manual therapy	63	Manual, simulated manoeuvre	21	33.3
		Manual, soft touch	19	30.2
		Manual, technique to different area	1	1.6
		Manual, simulated manoeuvre and to different area	1	1.6
		Disabled device	16	25.4
		Other (time-attention control, therapist attention, active joint movement, 2x low-pressure algometry)	5	7.9
Rehabilitation or physiotherapy	22	Exercise, nonspecific	8	36.4
		Exercise, key components altered	1	4.5
		Disabled device	5	22.7
		Manual, simulated manoeuvre	1	4.5
		Manual, soft touch	1	4.5
		Educational attention control	5	22.7
		Other (nonspecific visualisation, rest + therapist attention)	2	9.1
Cognitive-behavioural and other psychotherapy	27	Multicomponent therapist interaction	11	40.7
		Educational attention control	7	25.9
		Cognitive task, nonspecific	2	7.4
		Writing attention control	2	7.4
		Other (relaxing music, nonspecific visualisation, open-label saline injection, nonhypnotic relaxation suggestions and white noise, nonspecific video and sound)	5	18.5
Spiritual, energetic, or esoteric healing	8	Simulated hands-off manoeuvre (actor)	7	87.5
		Simulated hands-on manoeuvre (actor)	1	12.5
Self-management	5	Rest time control	2	40.0
		Educational attention control	1	20.0
		Web site, nonspecific	1	20.0
		Other (white noise)	1	20.0
Other	2	Other (white noise, headphones without sound)	2	100.0

N = 198.

### 3.4. Similarity between experimental and sham interventions

Conceptually, 29% of all studies explicitly reported matching or controlling for certain intervention components, but the degree to which sham control interventions resembled the tested intervention varied widely.

The average similarity between experimental and sham intervention per trial was 0.88 (SD ± 0.66) across all rated features. Assessment of individual features showed that some items were frequently designed to match the active intervention, while this was rare for others (Fig. 2, table with statistical detail provided in supplemental digital content 3, available at http://links.lww.com/PAIN/B673). For most items, however, confidence intervals were large. Overall ratings were different between simple and complex intervention trials (t(1,195) = 4.67, *P* < 0.0001), with comparisons between simple interventions and their shams being on average 0.4 points more similar (0.24-0.6 95% confidence interval).

**Figure 3.**
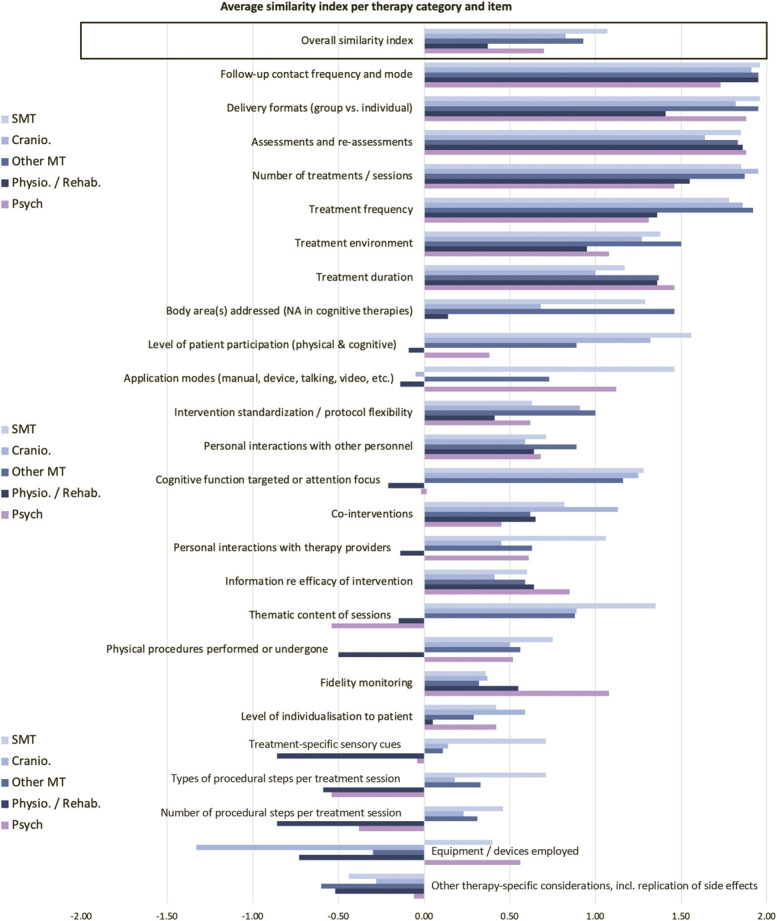
Average similarity ratings categorised by therapy type, comparing active and control interventions across 25 features. The overall mean across all items is provided in the top row. The end of each bar indicates the average rating. Measures of variability are provided in a supplementary table (supplemental digital content 3, available at http://links.lww.com/PAIN/B673) and the number of trials per group in the overview table 3.

Notable therapy-specific differences existed for overall similarity ratings (F(7,190) = 5.28, *P* < 0.001), with physiotherapy or rehabilitation trials having significantly lower average ratings (0.37 ± 0.77) than spinal manipulation trials (1.07 ± 0.54, *P* < 0.001), other manual therapies (excluding craniosacral therapy) (0.93 ± 0.6, *P* = 0.008), and trials of spiritual or energetic therapies (1.52 ± 0.42, *P* < 0.001). Figure 3 provides therapy-specific similarity ratings across all assessed features. A table with statistical detail is provided as supplement (supplemental digital content 3, available at http://links.lww.com/PAIN/B673).

### 3.5. Provider-related similarity

Interventions in the test and control groups were delivered by the same (set of) providers in at least 120 (59%) of all trials (clearly reported). Different providers were used in at least 32 trials (16%), and this could not be ascertained or was not relevant because of treatment automation in a further 46 (23%).

In trials where it was clearly reported that different providers were used, we further assessed whether these were matched for expertise (eg, educational background), experience (eg, years in practice), behaviour, and if trial-specific training had been similar between groups (Table 4).

**Table 4:** Matching of provider-related factors.

	Provider expertise		Provider experience		Provider behaviour		Trial-specific provider training	
	n	%	n	%	n	%	n	%
Matched	7	21.9	3	9.4	12	37.5	9	28.1
Not matched	18	56.3	16	50.0	5	15.6	9	28.1
Not reported	7	21.9	13	40.6	14	43.8	11	34.4

Assessed in trials for which it could be ascertained that different providers were used for active and control interventions. N = 32.

### 3.6. Additional features of placebo control interventions

Within the trials, methods of enhancing patients' expectations of a therapeutic effect included describing potential benefits of the sham intervention. Relatedly, some trials informed patients that only effective treatments were studied, either directly or by naming the sham control intervention differently (eg, a “physical modality”^105^). Providers' positive expectations were modified, for example, by not informing them that the sham device was disabled or by telling them that simple touch could have beneficial effects (Table 5).

**Table 5:** Additional features of placebo control design and implementation.

Feature	Reported in		
	n	%	
(Mis)informing patients that only efficacious treatments are investigated	32	16.2	
Deliberately enhancing patients' expectations of therapeutic benefit (other means)	16	8.1	
Only enrolling treatment-naïve patients	30	15.2	
Enhancing control *provider* expectations of therapeutic benefit	9	4.8	Not applicable in 10 automated interventions (n = 189)
Providing specific training to the therapists to deliver the control intervention	43	22.8	Not applicable in 10 automated interventions (n = 189)

Total n = 178, unless otherwise indicated. Note that the provided assessments are dependent on author reporting.

### 3.7. Sham control intervention development and theory

We examined the reporting of processes and theoretical considerations underpinning the development of each sham control intervention. Where reported, information on development processes or theoretical considerations was brief, often no more than a half sentence. Theoretical considerations included justifying why certain elements of a sham intervention were chosen or omitted. Overall, many studies provided no indication how the design of the control intervention was informed (Table 6).

**Table 6:** Reporting of placebo control development processes and theoretical considerations.

Sham control development	Reported in		Comment
	n	%	
Processes involved in the development of the sham control intervention	90	45.5	29 (14.6%) in the text and with some explanation
			61 (30.8%) provided a reference to previous work
Specific testing of the control intervention (including feasibility or validation phase, either as part of the trial or externally)	22	11.6	Not applicable in 8 validation studies (n = 190)
Consulting with patient, practitioners, or public involvement groups	4	2.0	
Consideration of ethical aspects of respective sham intervention	14	7.1	

Theory	Reported in	
	n	%
Theoretical considerations in the design of the sham control intervention	59	29.8	
Any hypothesized active (or specific) components of the treatment under investigation	160	80.8	
Any hypothesized nonspecific (or common) components of the treatment under investigation	58	32.6	
Matching or controlling of the nonspecific (or common) factors between therapy and sham	58	29.3	

Total n = 178, unless otherwise indicated.

### 3.8. Blinding

Assessing compliance with a relevant CONSORT reporting item, 53% of all included studies reported the blinding status of *all* involved stakeholders (patients, providers, outcome assessors, and statisticians). An additional 36% reported the blinding status for some of the above. Although trials were designed to blind patients to group allocation in 75% of cases, information on patient blinding was not provided in 13% of reports, and 12% of sham-controlled RCTs reported that the trial was not designed to blind participants to the nature of the intervention received or the group allocated to. Although trial reports were often ambiguous on the specific circumstances, it seems that in these instances, patients may have been aware of the group they had been allocated to, often because the 2 interventions were very dissimilar. They were, however, in most instances likely not aware that the control intervention was a sham control with no supposed effect on outcomes.^5,10,11,40,44,45,92,102,105,111,134^ Consequently, these trials were sham-controlled but used deception as to the nature of the comparator intervention. In other instances,^6,13,57,70,90,96,132,153,157,158^ control interventions were used that were not believed to be entirely inert but have circumstantial effects on outcomes. Almost exclusively, these latter were so-called attention controls for cognitive-behavioural interventions.

Providers were blinded in a minority of 3% of trials, but the methods to achieve double-blinding are noteworthy: Ajimsha et al.^3^ and Moraska et al.^116^ did not inform practitioners that the used ultrasound machine was nonfunctional. In another case, the control intervention was provided by family members who read to the patients, essentially providing an attention control without knowing about its rationale in the trial context.^51^ A similar strategy was applied to practitioners by Vitiello et al.,^152^ with providers delivering an educational attention control not knowing that it was the trial's control condition. In a further 7% of trials, provider blinding was of no concern because, for example, automated or prerecorded interventions were studied.

Blinded outcome assessment was reported for 58% of studies. A further 24% exclusively used patient-reported outcome measures, thus ensuring blinded outcome reporting where patient blinding was successful. Unblinded outcome assessment was reported in 6% of trials, and information on blinding status of outcome assessors was not reported in 11% of trials. The separation of treatment provider and outcome assessor roles was another common method to enhance internal trial validity, reported in 67% of trials (not performed in 6% and not reported in 28%).

Whether the statistical analysis was blinded was rarely reported (69% not reported), with 21% of trials reporting blinded, and 10% unblinded, statistical analysis.

### 3.9. Reports of blinding effectiveness and patient expectancy

Of 198 control interventions, 150 (76%) were most likely designed to blind participants to the received intervention. Only in 35 (23%) of these cases did researchers evaluate whether participants blinding had been successful, which included all but one of the 8 sham control validation studies. Blinding was mainly assessed by patients guessing their group allocation and occasionally through treatment credibility as proxy. The methods to analyse and interpret blinding success were highly variable.

In 19 reports, blinding indices were provided or data were reported in a manner that allowed for calculating Bang's index. Only 4 studies reported unsuccessful participant blinding as per their own criteria; all others reported successful blinding or provided descriptive data without judgement. Details and results are reported in a supplementary table (supplemental digital content 4, available at http://links.lww.com/PAIN/B674).

Two small cross-over studies assessed blinding effectiveness.^76,142^ In cross-over designs, patients can directly compare experimental and sham treatments, arguably making it easier to correctly guess group allocation. However, there was no indication of less successful blinding in the second phase of the trial by Hall et al.^76^ Teys et al.^142^ indicated successful blinding but did not provide useful data for independent assessment.

The time points of blinding assessment differed, with most trials obtaining ratings after the first session or after the end of the treatment. Few studies monitored blinding throughout the course of a longer trial.^47,104^ Notably, however, 22 other studies (11%) reported that their sham intervention had been tested previously.

Apart from reporting on blinding success, 29 studies (14.6%) assessed the patients' expectation of treatment benefit, albeit in a very heterogeneous manner, or their satisfaction with the received interventions (9 studies overlapping with those reporting on blinding success). Occasionally, this was reported as a proxy for successful blinding but more commonly to study potential influences of patient expectancy on clinical outcomes. Further detail is provided in supplementary digital content 2, available at http://links.lww.com/PAIN/B672.

## 4. Discussion

We analysed 198 sham control interventions and compared them with respective experimental treatments, identifying a range of common control intervention designs. We found notable gaps in reporting important information about the development, rationale, and validation of used sham controls, complicating the assessment of control intervention quality as well as the replication of methods by future researchers. Blinding effectiveness was also rarely reported and, if so, was performed in a variety of ways. The large and heterogenous sample studied here allows for a nuanced discussion of control and blinding methods in PPS trials for pain.

Based on the concepts of “structural equivalence” and “indistinguishability,”^21,108,125^ we provided a detailed assessment of the similarity between control and experimental interventions. In our sample, similarity was prioritised for features concerned with the extent and timing of treatments and outcome assessments and the delivery format. The environments in which control and experimental interventions took place were also similar on average, but the variability was larger and nonreporting contributed to lower ratings. Many other compared features were less commonly matched between groups. These concerned the patient experience (eg, treatment-specific sensory cues such as touch or sound, attention focus during interventions, personal interactions with providers and staff), procedural aspects of interventions (individualisation to patients, similarity and complexity of physical procedures performed, devices used in the application of control but not experimental treatments, use of cointerventions), and research-related aspects (eg, differences in fidelity monitoring). Furthermore, developing closely matched control interventions is less common for complex intervention studies.

### 4.1. Challenges of control intervention design

The findings illustrate the intricacies of designing adequate control interventions in efficacy and mechanistic trials. For example, the closer control interventions are matched to experimental treatments, the more challenging the necessary mechanistic considerations become. In manual therapy trials, concerns regarding the supposed inherent benefits of human touch^110^ may lead authors to consider nontouch control interventions. Interestingly, while massage-based or mobilisation-based treatments and craniosacral therapies are often compared with detuned ultrasound or other devices,^46^ the field of spinal manipulation research has opted against such an approach.^48,112,125,150^ Mechanistic studies of spinal manipulation have focussed on the “click” phenomenon and thrust forces.^75,123,124^ Contrastingly, in nonthrust techniques, the supposed mechanism is less clear-cut or more subtle, leaving more room for the potential role of touch.^26,27^ The use of actors was the preferred control intervention in RCTs of energetic or spiritual healing practices,^14,19,29,42,129,130,135^ likely again explained by mechanistic considerations where the healer themselves is the mechanism or medium through which healing occurs.^141^

Relatedly, in a trial of guided imagery for pain relief,^18^ the patient's attention focus on the breath and away from the pain experience is an integral part of the treatment and will thus not be matched. As such, it is unclear whether an optimal control should direct attention to something else non–pain-related (as would be the case in a general health education programme used as attention control) or not manipulate attention at all (as in the given example, using “rest” as sham control). The question of attention focus also applies to physical and manual therapy trials. Some control interventions involved treatment of or exercises for nonaffected body parts,^54,73,91,114,122,144^ producing a mismatch with the experimental treatment where patients were likely to focus on painful body areas.

In psychological intervention research, the complexity of treatment mechanisms has probably contributed to a relative sparsity of controlled efficacy or mechanistic trials. Instead, psychological interventions such as cognitive-behavioural therapy are often compared with treatment-as-usual or no-treatment controls,^61^ against which they show small to moderate effects.^166^ Existing studies with active comparators, few of which qualified as sham or attention controls in our review, only show very small effects on pain and disability.^166^ Indeed, “specific” (eg, behaviour change) and “common” (eg, the therapeutic relationship) treatment mechanisms are often linked and difficult to isolate in psychological interventions^156^ and elsewhere.^97,148^ As an alternative approach, mediation analyses have been used within trials of active psychological treatments to advance understanding of purported mechanisms of change.^118,145^

Although the challenges for sham-controlled psychological intervention trials are certainly immense, there are mechanistic theories that could guide control intervention development.^4,38,39,109^ Furthermore, our review demonstrates that high-similarity control interventions are feasible,^70,90,153^ likely providing more insight into treatment efficacy and mechanism than unmatched active comparator treatments such as education, relaxation, or exercise.^165^ In addition, many manual therapy^2,15,17,25,28,37,63,73,74,89,100,106,120,136,140,142,151,167,170^ and some exercise trials^69,84^ found promising solutions to the sham control problem, creating largely similar control interventions through the consideration of mechanistic treatment rationales and the mimicking of main contextual treatment aspects. This approach may in turn inspire development in other therapy fields, including psychological interventions.

The above examples of touch, attention focus, and active comparator treatments also illustrate another challenge of controlled efficacy RCTs in PPS research: It is unclear what the implications for a trial are if the used control intervention is considered a treatment in its own right under different circumstances, such as cognitive distraction, nonspecific exercise, generic education, provider support, or touch. Calling control interventions “sham” rather than “placebo control” acknowledges that these may not be as clearly “inert” as a sugar pill. Nevertheless, the question remains whether the effect sizes expected in pharmacotherapy research can realistically be demanded from sham-controlled RCTs of PPS interventions, given the potentially considerable effects produced by complex sham comparators.^61^

What constitutes an appropriate control intervention can be informed by placebo research^147^ and may depend on the trial's objectives. If the aim is to create similar levels of patient expectations of benefit, then studies need to explore whether this can be achieved with very dissimilar controls^68,127,169^ or even unblinded designs.^164^ If the aim is to control for context effects or study treatment mechanisms, then a careful matching is likely beneficial.^125^ Blinding to sham allocation alone may also be achieved with very dissimilar but equally credible interventions, as illustrated by 2 reviewed trials that assessed blinding success.^23,52^ However, this approach is unreliable,^155^ and blinding is likely helped by intervention similarity.^35^ Measuring potential outcome mediators such as expectancy and blinding status is laudable but uncommon and so is the testing of control interventions in pilot studies. In the absence of such information, readers of a trial can only put themselves into the patients' shoes and ask whether this control intervention would feel credible and effective to them.^83^ In our parallel publication, we further assess the impact of matched or nonmatched controls on trial outcomes and discuss potential “giveaways” that may undermine the blinding success of even well-designed control interventions.

For further inspiration for control interventions and examples from a given group of therapies, the reader is referred to the comprehensive supplementary table (supplemental digital content 2, available at http://links.lww.com/PAIN/B672) where each trial and its control design is categorised and to the supplementary table on reported blinding effectiveness (supplemental digital content 4, available at http://links.lww.com/PAIN/B674).

### 4.2. Additional blinding considerations

Blinding of treatment providers was very rare in the included trials (reported in 3%). Arguably, however, the potential for unblinded therapists to undermine participant and staff blinding is considerable and so is their capacity for producing different contextual effects between groups.^99^ Especially in studies where providers spend substantial time with patients, it seems reasonable to suspect that providers might “compensate” for providing control treatment by changes in behaviour and possibly additional advice or other contraventions of trial protocols. It is inherently challenging to achieve provider blinding, especially when a trial is delivered in a real-world clinical setting. However, unless nonblinded providers are prepared for situations in which their natural inclination to help might contravene trial requirements, a trial's internal validity is at risk.^99,107,165^

Where patient blinding to group allocation is an objective of the control intervention, the assessment of whether blinding was successful seems reasonable. In our sample, 25% of the relevant studies did examine this, some of which, however, were validation studies of new control interventions. Many of the recent arguments against such assessments and against blinding overall^9,162^ may not apply to the studied patient population and group of therapies. For example, unblinding because of dramatic treatment efficacy is unlikely in musculoskeletal pain and PPS interventions, and adverse effects are less common.^43^ The practical argument against blinding, however, namely that it may simply not be possible in such complex interventions,^162^ does warrant some consideration: This review has clearly shown that trial researchers and funders in pain research perceive there to be a need for sham-controlled and blinded trials, especially across the manual therapies. As Anand et al.^9^ rightly point out, there are research areas in which placebo effects are likely and where the case for the superiority of an intervention over a sham control has not yet been fully examined. On the other hand, emerging conflicting evidence regarding the impact of blinding status and blinding success warrants further scientific attention.^12,66,117^

The diversity and sometimes sophistication of used control interventions, plus the existence of multiple successfully blinded trials, demonstrates that patient blinding is a feasible, if challenging task. The complexity of the task, however, does lead to considerable research expense and, in the absence of best-practice standards for control interventions in efficacy and mechanistic trials, likely also research waste because of noncredible control interventions.^83^ Comparative effectiveness studies are the obvious alternative to sham-controlled RCTs in complex interventions, but their adequacy needs to be considered in the light of the research question, existing evidence of efficacy, and the availability of suitable active comparator treatments.^165^ Given the need for larger sample sizes in such trials, it further seems questionable whether these designs are always more economical than a well-designed explanatory RCT.^16,82^

### 4.3. Reporting

Insufficient reporting of blinding methods has been identified as a problem before and has not seemed to improve.^8,72,163^ Recently, a checklist specific to the reporting of placebo controls was published (Template for Intervention Description and Replication [TIDieR]—Placebo).^86^ Although not formally assessed in our review because data extraction was completed before the publication of TIDieR-Placebo, we suspect that most procedural items of the reporting checklist are complied within PPS trials (what was provided as part of the control intervention, through which delivery modes, when and how much; items 1, 3, 4, 6, 8, and 9). However, we showed the reporting of provider characteristics to be deficient in trials where control and active interventions were not delivered by the same set of providers. Notably, TIDieR-Placebo requires little information on provider behaviour (only expertise, but not potential behaviour matching), an element for which we identified a large need for improved reporting. As for the theoretical background and rationale of the control intervention, TIDieR-Placebo asks researchers to “[d]escribe any rationale, theory, or goal of the elements essential to the placebo/sham intervention.” We were able to ascertain that information to this effect was only provided in about a third of the studies. Even so, this was rarely sufficient to understand the relevant theoretical considerations regarding the control intervention design, including the purpose of using a sham control in this specific trial (blinding to group allocation, controlling for contextual effects, both), or to isolate the specific treatment components of the experimental treatment. Knowing the trial authors' reasoning allows readers to assess the appropriateness of the control intervention.^165^

While reporting guidance for intervention components only became available in 2014,^80^ reporting guidelines for general trial features have been available longer. Specifically, the 2008 publication of the first CONSORT statement for nonpharmacological intervention RCTs^30,32^ is the reason why we included studies published from then onwards. Irrespectively, reporting of the 2 major items relevant to this review's objectives—the detailed description of the control intervention (66%) and reporting of the blinding status of all involved stakeholders (51%)—requires some improvement.

## 5. Conclusions

Overall, our findings call attention to the need for more guidance on the design of control interventions and blinding methods in mechanistic and efficacy trials, informed by current practice and common challenges in the field of psychological, physical, and self-management intervention research. Currently, sham controls range from closely resembling the test treatment to highly dissimilar, with differences between therapy groups. Especially, physiotherapy and certain kinds of manual therapies use dissimilar controls. Despite being a primary objective of most sham control interventions, it is infrequently reported whether participant blinding was effective.

Future recommendations for sham control interventions need to begin with a consideration of whether a sham-controlled RCT is the adequate design for a given research question and, if so, what the phenomena to be controlled for are. Control intervention development is likely improved by being theory-driven. In this context, insights from placebo research may be useful and we examine the link between sham similarity and trial outcomes in a second publication of this review.^81^ Feasibility testing may be helpful to ascertain whether a control intervention can achieve its objectives. To be useful for end users, the reporting standard of control procedures needs to be enhanced.

While the complexity of the task may mean that research efforts cannot be directly compared with pharmacological RCTs and that alternative designs may have to be considered, our review clearly demonstrated the feasibility of successful blinding by means of dedicated complex control interventions in large-scale RCTs of PPS therapies.

## Conflict of interest statement

Mr Hohenschurz-Schmidt reports support through a PhD Studentship from the Alan and Sheila Diamond Trust for this work and personal fees from Altern Health Ltd, outside the submitted work; Dr. Draper-Rodi reports grants from Alan and Sheila Diamond Charitable Trust, during the conduct of the study; Dr. Scott reports grants from Medical Research Council and Versus Arthritis, and from the National Institute for Health and Care Research, outside the submitted work; Dr. Vollert reports personal fees from Vertex Pharmaceuticals and personal fees from Embody Orthopaedic, outside the submitted work; Prof Rice reports personal fees from IMMPACT and grants from the Alan and Sheila Diamond Trust during the conduct of the study, and personal fees from Imperial Consultants, personal fees from MD Anderson Cancer Center, other from spinifex, other from Medicines and Healthcare products Regulatory Agency (MHRA), and Commission on Human Medicines - Neurology, Pain & Psychiatry Expert Advisory Group, all outside the submitted work; In addition, Dr. Rice has a patent WO 2005/079771 & a patent EP13702262.0/ WO2013 110945 pending. All other authors report that they have no conflicts of interest.

## Appendix A. Supplemental digital content

Supplemental digital content associated with this article can be found online at http://links.lww.com/PAIN/B671, http://links.lww.com/PAIN/B672, http://links.lww.com/PAIN/B673, and http://links.lww.com/PAIN/B674.
